# Escape from X-chromosome inactivation: from gene discovery to regulatory mechanisms

**DOI:** 10.1042/BST20250104

**Published:** 2026-07-09

**Authors:** Caterina Simoncini, Agnese Loda

**Affiliations:** 1 Institut *Imagine*, Inserm UMR 1163, 24 Boulevard du Montparnasse, 75015 Paris, France; 2 Institut Pasteur, Department of Developmental and Stem Cell Biology, 25-28 Rue du Dr Roux, 75015 Paris, France; 3 Université Paris Cité, 45 Rue des Saints-Pères, 75006 Paris, France

**Keywords:** chromatin dynamics, Developmental gene regulation, Dosage Compensation, Sex Differences, X-chromosome inactivation

## Abstract

Early in mammalian development, one of the two X chromosomes in female embryos is largely silenced through X-chromosome inactivation (XCI). Although essential for dosage compensation, XCI is incomplete, with ∼5%–20% of X-linked genes escaping silencing. Escape from XCI represents an important source of sex-biased gene expression and has been increasingly linked to sex differences in development and disease susceptibility. Yet, how a subset of X-linked genes bypass XCI remains poorly understood. Here, we discuss the studies that have revealed the prevalence and variability of XCI escape across genes, tissues, and individuals in both humans and mice. We then summarize current insights into the molecular features and regulatory mechanisms associated with XCI escape, highlighting key questions that remain to be addressed to understand how X-linked gene dosage is regulated and how it contributes to sex-biased biology.

## Introduction

In mammals, dosage compensation of X-linked gene products between females (XX) and males (XY) is achieved by X-chromosome inactivation (XCI), the epigenetic process triggered by the X-linked non-coding gene *Xist* [1]. XCI results in the near-complete silencing of all but one X chromosome in XX females, as well as in individuals carrying supernumerary X chromosomes (e.g., 47,XXY; 47,XXX; 48,XXXY) [2,3]. In all these cases, XCI allows embryos with additional X chromosomes to survive throughout development, explaining why sex-chromosome aneuploidies are the most common and best-tolerated aneuploidies in humans [4]. Once established during early embryonic development, the silent state of the inactive X chromosome (Xi) is generally stably maintained through cell division in somatic tissues [5,6], but becomes reactivated in two developmental contexts: in cells of the mouse blastocyst that will give rise to embryonic tissues, where imprinted XCI of the paternal X chromosome (Xp) is reactivated before the onset of random XCI in the epiblast [7,8], and in female primordial germ cells, as recently reviewed by Rodriguez and colleagues [9].

**Figure 1:**
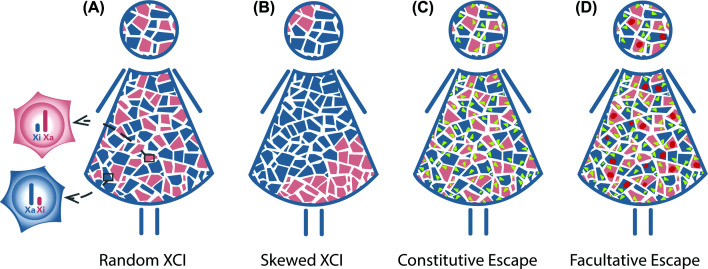
Females are mosaics for X-linked traits Schematic representation of cellular mosaicism in female mammals resulting from XCI and escape. (**A**) In random XCI, the maternal (pink) and paternal (blue) X chromosomes have an equal probability of being inactivated in each cell, generating a mosaic cell population composed of approximately equal proportions of cells with an active maternal or paternal X chromosome. As a consequence, both parental alleles of X-linked genes are equally represented across cells in the organism. (**B**) Skewed XCI is characterized by an unequal representation of cells expressing the maternal and paternal X chromosomes within a cell population. This can result either from genetic variation or mutations that bias the choice of which X chromosome is inactivated (primary skewing), potentially leading to complete non-random inactivation of the same X chromosome in all cells, or from cell selection following an initially random XCI event (secondary skewing). In the latter case cells expressing one X chromosome have a growth and/or survival advantage over cells expressing the other. The contribution of escape from XCI to X-linked mosaicism is illustrated for (**C**) constitutive and (**D**) facultative escape. Constitutive escapees (green) are transcriptionally active from both the Xa and Xi in the large majority of cells and tissues, resulting in consistent biallelic expression. Facultative escapees (red) evade XCI variably across individuals, tissues, and cell types; consequently, some cells express these genes biallelically, whereas others express the same genes monoallelically.

As a result of XCI, female mammals are therefore mosaics of X-linked traits composed of two different cell populations, each expressing either the maternal (Xm) or the paternal (Xp) X chromosome [10]. Because the two alleles are often not inactivated in a strict 50:50 ratio, the degree of cellular mosaicism varies across tissues and individuals, directly contributing to phenotypic variability [6,11,12]. In the context of X-linked mutations, for example, differences in XCI skewing from the 50:50 ratio can strongly influence both disease manifestation and severity by modulating the relative expression of mutant versus wild-type alleles [10,13] (Figure 1). Importantly, although XCI silences the vast majority of X-linked genes, a subset of genes escapes XCI and is biallelically expressed from both X chromosomes within the same cell [14] (Figure 1). This biallelic expression introduces yet another layer of cellular mosaicism; it often contributes to sex-specific gene expression and is expected to have a profound impact on sexual dimorphism in health and disease [2,13]. For example, several X-linked genes involved in immune function escape XCI, and their increased expression has been implicated in female-biased autoimmune diseases, with individuals carrying two X chromosomes (XX females and XXY males) showing a higher susceptibility to autoimmunity than XY males [13,15–17]. Furthermore, the biallelic expression of a subset of tumor suppressor escapees, known as escape from X-inactivation tumor suppressor genes (EXITS), has been proposed to protect females against certain cancers, as complete loss of gene function requires mutations in both expressed alleles, whereas males carry only one active copy that can be inactivated by a single mutational event [18]. However, despite the clear biological relevance of XCI escape, we still know remarkably little about how X-linked escapees resist epigenetic silencing by Xist RNA, as well as the exact causality between the expression levels and protein dosage of X-linked escapees and their impact on sex-biased phenotypes during development and throughout life. In the present review, we first provide an overview of which genes escape XCI and then discuss what is known about their regulation during and after XCI, highlighting the fundamental unresolved questions and unexplored hypotheses that remain to be tackled to move the field forward.

## Exceptions to the X inactivation rule

Mary Lyon was the first to propose that a certain level of gene activity from the Xi could explain the phenotype of Turner syndrome (45,X; ∼1/2000 births) and Klinefelter syndrome (47,XXY or 48,XXXY; ∼1/650 births) [19]. In the first case, the absence of the Xi results in loss of ∼99% of XO embryos *in utero* [20–22]. The very few women who survive with only one X chromosome are very often mosaics of XX and XO cells and frequently present with premature ovarian failure, infertility, cardiovascular and neurological abnormalities, and short stature [20–22]. Klinefelter syndrome is associated with male infertility and is characterized by tall stature and deficits in attention and social skills, although the condition remains undiagnosed in up to 70% of cases [23]. Lyon predicted that these phenotypes are likely the result of either the loss or duplication of a ‘short pairing segment’ of the X chromosome that is normally not inactivated [19]. The pairing segment proposed by Lyon in 1962 corresponds to the X–Y pseudoautosomal region (PAR) [24], a homologous region shared by the X and Y chromosomes that undergoes pairing and recombination during male meiosis to ensure proper sex chromosome segregation [25,26]. The human X chromosome contains two PARs, an exception among eutherians [27]. PAR1 spans ∼2.7 Mb at the distal end of the short arm of the X chromosome and contains at least 25 coding and non-coding genes [26,28]. All PAR1 genes tested so far escape XCI, including *SHOX*, for which a dosage effect is well documented. Haploinsufficiency of *SHOX* was shown to lead to the short stature characteristic of Turner syndrome, whereas additional copies are associated with increased height in Klinefelter syndrome [29–31]. Another PAR1 gene with a proposed dosage effect is *CSF2RA*, whose haploinsufficiency has been associated with abnormal placental differentiation and may contribute to the high early lethality of XO embryos, although direct causality has not been demonstrated [32]. PAR2 is much smaller (∼0.3 Mb), is located at the end of the long arm of the X chromosome, and contains four genes that display distinct XCI patterns: two escape XCI and two are subject to inactivation. Interestingly, the Y-linked homologs of PAR2 genes that are subject to XCI are also inactive [33,34].

The mouse PAR is the smallest described in eutherians and exhibits a high degree of sequence divergence and considerable differences in size across different inbred strains (i.e., ∼300–700 kb) [35–37]. Recent studies identified ten genes and four pseudogenes in the mouse PAR, revealing a higher degree of homology with human PAR1 than previously anticipated, although the majority of PAR1 orthologs are not located in the mouse PAR but either map to autosomes or are missing [26,36–38]. Because the mouse PAR has long been one of the most challenging genomic regions to sequence, the transcriptional activity of genes located in this region on the Xi is mostly undetermined.

Since Lyon’s original hypothesis, it has become clear that escape from XCI extends far beyond the PAR. Several clusters of escapees have been identified outside PAR1 in humans [39–41], and on the mouse X chromosome escapees are found both in clusters and as isolated active genes [42–58]. The number of identified escapees has expanded considerably over time and continues to grow, driven in large part by advances in the methods used to study XCI, which have led to a more comprehensive understanding of the prevalence and diversity of escape from XCI, as we discuss below.

## Gene activity on the silent X chromosome

An X-linked gene is considered to escape XCI when it is biallelically expressed in female cells [40]. Because expression from the Xi is typically reduced compared with the one from the active X chromosome (Xa) and rarely reaches equal allelic levels, a threshold of ∼10% expression from the Xi relative to the Xa is classically used as a cutoff to define escape, even if different thresholds have been used across different studies [59]. Escape from XCI has been studied mainly in mouse and human systems, which require distinct experimental strategies as reviewed by Peeters and colleagues [59]. Mouse models provide a major advantage, as the allele-specific expression of X-linked genes from the Xi can be directly detected in cell lines or tissues derived from F1 hybrid mice in which the two X chromosomes can be distinguished at the genetic level thanks to the high density of polymorphisms between inbred strains [45,48,49]. Moreover, targeted mutations in *Xist* or its regulators can be used to skew XCI, ensuring controlled inactivation of the same X chromosome across cells [43,60,61]. This allows measuring the levels of Xi expression at the cell population and tissue level, rather than requiring single-cell analyses. Detecting escape in human cells and tissues is more challenging. Information on genetic variation between the two X chromosomes is often unavailable, and although complete skewed inactivation of the same X chromosome occurs in some women [41,62], XCI is typically random or only partially skewed in human tissues, requiring single-cell technologies to detect escape at allelic resolution. These approaches are often limited by the low expression levels of escapees and the sparsity of single-cell data; however, rapidly growing computational tools are improving their power, as discussed below [41,62–65].

The first comprehensive analysis of XCI escape in human cells was performed using mouse–human somatic cell hybrids carrying either the human Xa or Xi, enabling the classification of X-linked genes as monoallelically or biallelically expressed [40]. This seminal study revealed that at least 15% of X-linked genes consistently escape XCI, and it uncovered an unexpectedly high degree of variability among females, with an additional ∼10% of genes displaying heterogeneous escape across different hybrids. Another strategy used to investigate XCI escape in human contexts is DNA methylation profiling. Comparative analyses of XO and XX samples revealed that escapees are characterized by lower promoter and higher gene body methylation compared with silenced genes, leading to the use of these profiles as robust indicators of escape [66–69]. Integration of data from somatic cell hybrids and DNA methylation profiling confirmed that ∼25% of X-linked genes can evade XCI, often variably [39]. To assess the degree of tissue-specific escape within the same individual, allele-specific RNA-seq analysis was performed across 16 tissues obtained from a female donor with fully skewed XCI [41]. Up to 23% of X-linked genes were confirmed to escape XCI, consistent with previous reports, and ∼6% of these genes were shown to resist XCI in only one tissue, revealing a largely unexplored level of context-dependent variability within the same individual [41]. The analysis of samples from two newly identified women with skewed XCI in the Genotype-Tissue Expression (GTEx) database extended the investigation to 15 additional tissues and showed that tissue-specific escape occurs for 11% of X-linked genes [62]. Variability of escape across tissues was also assessed in a large cohort of twins with skewed XCI, showing that 16%, 26%, and 29% of X-linked genes escape XCI in lymphoblastoid cell lines, adipose tissue, and skin, respectively, with 30% of these genes escaping in only one of the analyzed tissues [70]. The analysis of samples with skewed XCI facilitates the study of escape using bulk RNA-seq, as it simplifies the assignment of allelic expression to the Xa or Xi. However, skewed XCI typically reflects either primary skewing, caused by mutations in XIST or its regulators and leading to non-random XCI, or secondary skewing, resulting from selection against deleterious X-linked mutations or X–autosome translocations [6], both of which may influence the developmental dynamics of XCI in the embryo. Whether this affects the establishment or extent of escape remains unclear.

The direct assessment of XCI escape in cells with random XCI can be performed using single-cell RNA-seq [41,62–65]. A recently developed strategy based on pseudobulk and single-cell-level differential gene expression analysis quantified Xi gene activity across more than 1.5 million immune cells from over 600 individuals [65]. The present study revealed cell-type differences in escape, with higher Xi expression in lymphocytes than in monocytes, confirming previous observations in identical twins [70]. Similarly, the analysis of a multi-organ dataset from 6 women across 19 organs showed stronger escape in lymphatic tissues, although in this case, conclusions on cell-to-cell escape variability within the same tissue were limited by sample size [65]. This observation is consistent with studies in both humans and mice showing that naïve B and T lymphocytes exhibit incomplete maintenance of XCI due to loss of XIST/Xist localization on the Xi and alterations in Xi-associated chromatin features, resulting in increased expression of X-linked immune genes and potentially contributing to female-biased autoimmunity [71–74]. Single-cell transcriptomic methods also confirmed that female-biased expression levels of X-linked genes are often indicative of escape from XCI, as they can reflect transcription from the Xi. In the case of *KAL1*, for example, this gene displayed strong female-biased expression in the lung, the only tissue in which it evades XCI [41]. However, across studies, not all female-biased genes were shown to escape XCI, and sex differences in expression should therefore be interpreted with caution, as they may result from mechanisms independent of escape, such as hormonal effects.

In contrast with humans, where different approaches consistently estimate that up to ∼25% of X-linked genes escape XCI, a comparative analysis across 12 mammalian species identified the mouse as an outlier, with only ∼5% of X-linked genes escaping XCI [75]. Allele-specific RNA-seq of mouse tissues with skewed XCI confirmed escape of 3%–7% of X-linked genes [45,55,60], whereas studies of tissues with random XCI, including the same organs, reported escape of more than 20% of X-linked genes [42]. Although these discrepancies likely rely on the strategies used to infer escape and the difference between random and non-random XCI, escape levels ranging from ∼4% to ∼20% have been described in immune cells, (extra)embryonic tissues, and clonal cell lines such as kidney Patski cells and neural progenitor cells (NPCs) derived from mouse embryonic stem cells (ESCs) differentiation [43,45,46,48–50,57,61,76].

Similarly to the somatic hybrid system that was instrumental in revealing variable XCI escape in humans, the generation of mouse–rat allodiploid cells by fusion of haploid embryonic stem cells led to the discovery of more than 100 new escapees on the mouse Xi, 29 of which have human orthologs that also escape XCI, highlighting the conservation of escape across species [50]. In the case of *in vitro*-derived NPCs, subcloning single cells from a heterogeneous cell population with random XCI enables the assessment of cell-to-cell variability in escape, with independent clones showing escape of 40 to over 130 genes along the Xi [48,76,77]. Whether this level of cell-to-cell variability reflects the *in vivo* situation remains unresolved. However, heterogeneity of escape for a subset of these genes has been demonstrated by RNA-FISH *in vivo* [48,78–81], and a comparison between escapees identified in NPCs and those reported in 19 studies assessing escape in different contexts showed that out of ∼850 X-linked genes examined, ∼25% evade XCI in at least one case [76]. This suggests that escape from XCI exhibits greater tissue- and cell-type specificity than previously appreciated, even in the mouse. Consistent with this, a recent *in vivo* study using low-input bulk RNA-seq of four sorted cardiac cell types identified cell-type-specific escapees that were not detected in whole-heart analyses [60].

## Different patterns of X-linked escape

It is becoming increasingly recognized that the extent to which a gene escapes XCI likely reflects why it escapes and how its activity is regulated, both questions that are the object of active research. In the case of escapees referred to as ‘constitutive’, these genes seem to evade XCI from the onset of the process, showing little or no evidence of silencing during development and across the majority of tissues examined [76,80]. Their escape status is often conserved across species, and they are major contributors of sex-specific gene expression, as they are typically expressed at higher levels than other escapees on the Xi [2,82,83]. The majority of constitutive escapees are highly dosage-sensitive genes belonging to ‘X–Y pairs’ of homologous genes that resisted decay on the Y chromosome during the evolution of the sex chromosomes from an ancestor of autosomes [84,85]. These genes participate in processes that are essential for development and tissue homeostasis, including transcription, translation, RNA splicing, and chromatin dynamics. As a consequence, their dysregulation is frequently associated with developmental lethality and disease, often in a sex-biased manner [85,86]. Many constitutive escapees function as components of multiprotein complexes in which even small changes in protein abundance can have broad downstream effects [85,87–89]. This further explains their dosage sensitivity and their selective pressure to resist XCI, ensuring tight regulation of their expression and protein levels.

On the human X chromosome, 12 escapees belong to X–Y gene pairs, including the histone demethylases KDM6A and KDM5C, the transcription factor (TF) ZFX, the translation initiation factor DDX3X, and the deubiquitinase USP9X [84,87]. Interestingly, these escapees are located outside the PARs and may therefore have functionally diverged from their Y-linked homologues, leading to the emergence of sex-specific functions of X–Y protein pairs both in humans and mice [85,87,90]. For example, loss of *Kdm6a* in mice is embryonic lethal in females, whereas only ∼20% of mutant males survive to adulthood, indicating that the Y-linked homologue *Kdm6c* provides only partial rescue and suggesting functional divergence between the two proteins [91,92]. In general, Y-linked homologues appear to have evolved male-specific functions, particularly in spermatogenesis [85,87]. However, whether X-linked escapees have acquired additional extracatalytic activities beyond their ancestral catalytic functions, and how their increased dosage contributes to sex-specific phenotypes in gonadal and non-gonadal tissues, is largely unexplored. Nevertheless, a growing body of evidence supports the role of constitutive escapee dosage as a determinant of sex differences. For example, higher expression of *Kdm6a* in XX relative to XY individuals has been shown to contribute to female protection from bladder cancer, with loss of *Kdm6a* increasing cancer susceptibility [93]. Increased *Kdm6a* dosage has also been implicated in resilience to cognitive decline and Alzheimer's disease-associated pathology [94]. Consistent with a broader role in brain function, elevated *Kdm6a* expression enhances learning and memory in aging XY male mice, independently of its demethylase activity [95]. In the immune system, elevated *Kdm6a* expression resulting from escape from XCI contributes to sex differences in autoimmune responses and has been linked to the increased susceptibility of females to multiple sclerosis [96]. Likewise, *Kdm5c* dosage has been identified as a determinant of sex differences in adiposity, with reduction of *Kdm5c* dosage in XX mice to male-equivalent levels reducing body weight and fat mass [97].

**Figure 2:**
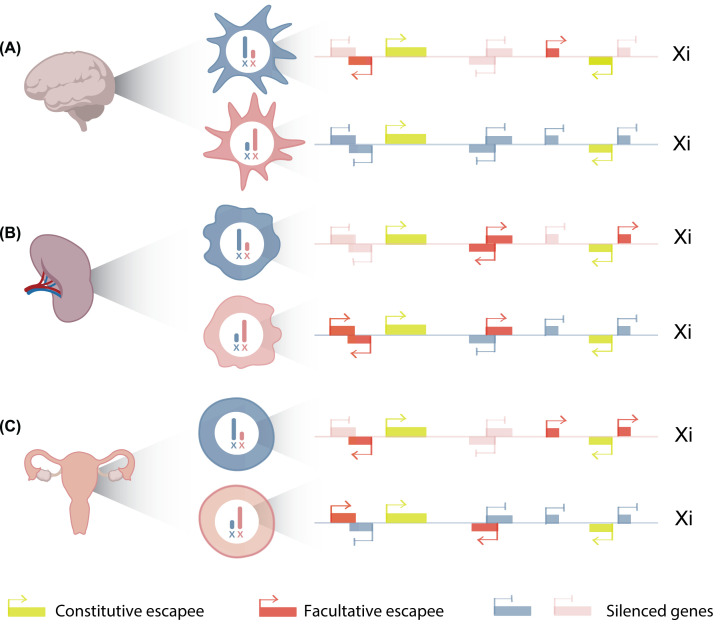
Facultative escape is variable across tissues and cells Schematic representation of facultative escape from XCI in (**A**) brain, (**B**) spleen, and (**C**) uterus. In each tissue, individual cells randomly inactivate one of the two X chromosomes, resulting in cells carrying either the maternal (pink) or paternal (blue) Xi. Gene activity from the Xi is illustrated for each tissue. Constitutive escapees (green) are largely expressed from the Xi across tissues and cells. Facultative escapees (red) show variable expression from the Xi: the same gene may be expressed in some cells but remain silenced in others. As a consequence, their activity is heterogeneous between tissues and also between cell types within the same tissue.

On the other hand, the very large majority of genes that escape XCI are likely not under strong selective pressure as they have lost their functional Y homologues and display highly context-dependent escape, being actively transcribed on the Xi only in specific tissues or cellular states and often varying across individuals [14] (Figure 2). The reasons why these ‘facultative’ escapees evade XCI remain unclear. Whether their transcriptional activity on the Xi results from a defined regulatory program or instead reflects unstable maintenance of XCI is being increasingly investigated, together with the functional consequences of their higher expression levels in females compared with males. Emerging evidence shows that facultative escape may be either neutral, advantageous, or deleterious, with accumulating examples suggesting context-dependent outcomes for female development and health. For example, facultative escape of *Atrx* was shown to rescue female mice carrying an *Atrx* mutation from the embryonic lethality caused by loss of *Atrx* in the extraembryonic compartment [80,98]. Furthermore, aging-driven escape and up-regulation of *Plp1* in the female hippocampus were shown to improve learning and memory in aged mice [99]. Because *PLP1* levels are increased in the aging hippocampus of women, its higher dosage in females may in fact contribute to sex-biased resilience to cognitive decline [99]. Similarly, age-specific escape of the *Ace2* gene has been reported in the lung, where it was shown to confer protection against pulmonary fibrosis, a condition that is more prevalent in men, suggesting that higher *Ace2* expression in females may be beneficial [60]. Other examples linking variable levels of escapees to sex-biased disease predisposition come from autoimmunity, where nearly 80% of patients are women [15,100,101]. This bias has been shown to result from the higher dosage of immune-related genes that escape XCI or become reactivated on the Xi [15,100]. For example, in the case of the facultative escapee *TLR7/Tlr7*, its increased dosage has been shown to predispose to systemic lupus erythematosus (SLE) in both humans and mice [102,103]. In mice, *Tlr7* deficiency protects against SLE-like symptoms, whereas males carrying additional copies of *Tlr7* spontaneously develop the disease [104,105]. More recently, the facultative escape *SMC1A*, a cohesin complex subunit involved in genome organization, has also been implicated in SLE. Increased *SMC1A* dosage promotes the expression of immune and inflammatory genes, suggesting a potential contribution to the strong female predominance of the disease [106]. Finally, another example of deleterious facultative escape is the case of *MED14* in human mammary stem cells, where its increased dosage stabilizes the Mediator complex, perturbing stem cell homeostasis and enhancing the tumorigenic potential of cells [107].

The list of escapees for which dosage changes affect tissue homeostasis is rapidly increasing. An important open question is whether the biological impact of escape primarily reflects the activity of individual dosage-sensitive escapees in specific cellular contexts or the broader consequences of increased transcriptional activity across the Xi as a whole, with both mechanisms likely contributing to different extents depending on the biological context. In addition, the inactive X chromosome itself may contribute to sex differences independently of escapee dosage through mechanisms linked to its unique heterochromatic organization and interactions with the nuclear environment. These effects may be influenced by Xi dosage, which varies not only between XX and XY individuals but also across sex chromosome aneuploidies.

## How some X-linked genes escape XCI is largely a mystery

**Figure 3:**
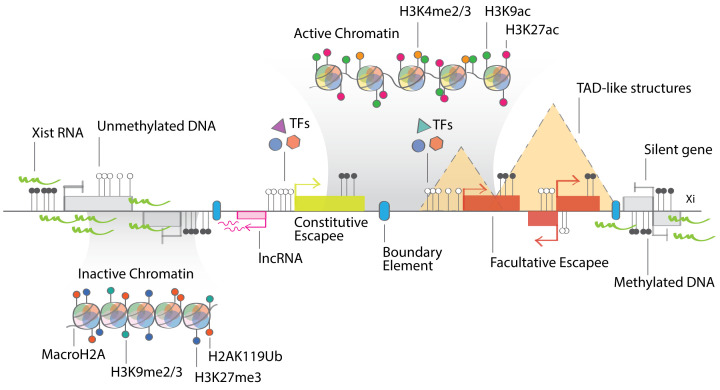
Mechanisms regulating escape from X-chromosome inactivation Schematic representation of active and inactive chromatin domains on the Xi, showing genes subject to XCI and escapees, together with putative escape regulators. Silenced genes (gray) reside within inactive chromatin domains and display features of transcriptional repression, including promoter CpG island methylation, enrichment of repressive histone marks and variants (H3K27me3, H2AK119ub, macroH2A, and H3K9me2/3), and Xist RNA coating. Escapees are located within active chromatin domains enriched for H3K4me2/3, H3K9ac, and H3K27ac histone marks. Both constitutive escapees (green) and facultative escapees (red) exhibit unmethylated CpG islands at their promoters, gene body DNA methylation, and increased chromatin accessibility. Proposed or identified mechanisms contributing to escape regulation are illustrated, including binding of XCI-resistant TFs at promoter-proximal regions, *cis*-acting DNA boundary or insulator elements, TAD-like chromatin structures associated with clusters of facultative escapees, Xist RNA levels, and regulatory long non-coding RNAs.

Most of what we know about the regulation of escapees comes from the characterization of their chromatin landscape on the Xi, including histone modifications, chromatin accessibility, Xist occupancy, and 3D structural organization. When expressed, escapees tend to localize outside the Xi territory coated by Xist RNA, and their promoters retain histone marks associated with active transcription and lack DNA methylation [108,109]. Furthermore, regions of active transcription on the Xi are not enriched in repressive histone marks deposited by the polycomb repressive complexes typical of silent genes [74,76,110], and clusters of facultative escapees are organized into topologically associating domain (TAD)-like structures, whereas the rest of the Xi largely lacks TAD organization [77] (Figure 3). Whether these chromatin and structural features of escaping loci on the Xi reflect their transcriptional activity or directly contribute to their regulation at the onset of XCI or later on, in adult tissues, is not fully understood. During mouse embryonic development, some facultative escapees were shown to be initially silenced and later reactivated, indicating that escape from XCI can be established dynamically [80], but whether this behavior is common among facultative escapees and whether there are specific factors driving their reactivation has not yet been determined.

One locus that has been relatively well studied as a model to understand escape regulation is the region encompassing the constitutive escapee *Kdm5c* [111,112]. Transgenic BACs of different sizes encompassing *Kdm5c* and its flanking genes were integrated at various locations on the X chromosome to detect genetic elements sufficient for escape. This strategy identified different genetic elements, including gene-proximal elements that allow *Kdm5c* to intrinsically escape XCI regardless of genomic context and a distal boundary element that prevents the ectopic escape of neighboring genes from adjacent X-inactivated regions [111,112]. The importance of gene-proximal elements in driving XCI escape is also supported by the integration of human BACs carrying either escapees or genes that are subjected to XCI at the same genomic location on the mouse X chromosome [113–115]. These experiments showed conservation of regulatory networks between humans and mice and indicated that regions of ∼2.6–6 kb spanning the promoter and part of the gene body of *KDM5C* and *RPS4X* are sufficient to enable their escape [59,114,115].

Promoter-proximal regions within ∼5 kb upstream of transcription start sites of escapees were also identified as the most accessible regions on the Xi by ATAC-seq chromatin accessibility analysis in female cells [77]. Similar results were obtained in human T cells by comparing chromatin accessibility profiles between female and male cells, showing that putative regulatory elements are more frequently located at promoters or within introns of escapees [116]. Although these studies point to an important role for promoter-proximal elements in escape regulation, how these elements function is not well understood. A step forward in this direction has been the identification of candidate trans-acting factors that bind these regulatory elements through analyses of enriched TF-binding motifs [114,117]. For example, the binding motifs of YY1, TCF7, FOXC1, and ATF3 were found to be overrepresented at escapee promoters in large-scale human datasets, although evidence of binding was confirmed only for YY1 [117]. More recently, YY1 binding on the Xi has been shown to act as a barrier to Xist-mediated silencing, persisting at regulatory elements of X-linked genes that undergo slow silencing during XCI and thereby delaying their inactivation. Importantly, although these genes eventually become silenced in this experimental setup, many are facultative escapees in other contexts, supporting a more direct role for YY1 in promoting escape [118]. Additional putative regulators were predicted by comparing ChIP-seq binding profiles at promoters of female and male datasets, identifying factors such as PAX5, MYC, TBLR1, FOXM1, MAZ, ATF2, CTCF, CHD1, and SIN3A [117]. More recently, enrichment analyses of TFs binding motifs across a set of 55 human escapees identified five top escape-enriched TFs (i.e., ZFP36, NIPBL, MYB, STAT1, and HSF1) [114]. However, in the case of *RPS4X*, its promoter contains binding motifs for all these five TFs, but contrary to expectations, promoter-proximal elements alone are not sufficient to recapitulate its escape from XCI in a reporter assay [114]. These results suggest that additional regulatory elements located in the downstream gene body are required [114]. However, the causal roles of most identified TFs in escape regulation remain to be experimentally tested.

One TF that has been proposed to act at multiple levels in the regulation of escape is CTCF [45]. In fact, CTCF not only binds promoters of escapees in a manner that depends on their XCI status [45], but is also enriched at intergenic regions between escapees and neighboring silent genes, where it may contribute to the establishment of insulating boundaries that separate active and inactive chromatin domains on the Xi [45,119,120]. Although targeting multiple CTCF binding sites near an X-linked reporter was not sufficient to drive escape or prevent the spreading of heterochromatin [121], deletion of a CTCF binding site at the endogenous boundary between the facultative escapee *Car5b* and its silent neighbor *Siah1b* resulted in loss of *Car5b* escape [122]. These observations suggest that the insulating function of CTCF at escaping loci may be locus-specific. Furthermore, CTCF plays an important role in organizing 3D chromatin architecture and may regulate escape by contributing to the TAD-like organization of clusters of escapees on the Xi [77]. In differentiated cells, transcriptional silencing of clusters of facultative escapees results in loss of TAD-like organization at these loci, suggesting that the 3D topology of the Xi reflects its transcriptional activity [76]. However, whether CTCF contributes to the establishment of this 3D structure at the onset of XCI, potentially providing a framework that protects escapees from silencing or helps maintain and restore their activity during and after XCI remains unclear.

The activity of long non-coding RNAs located near escapees has also been proposed to contribute to escape regulation. X-linked long non-coding RNAs showing female-biased expression were mapped near the constitutive escapees *Ddx3x*, *Eif2s3x*, and *Kdm5c* [51,52]. These loci lack enrichment of the repressive histone mark H3K27me3 and escape XCI, suggesting that small domains containing an escapee and a neighboring non-coding gene may form local units of escape regulation [51,52]. This may occur through active transcription that maintains an open chromatin environment near escapees or through a direct role of the transcripts themselves, although their precise contribution remains speculative. More recently, Xist RNA was shown to directly regulate escapee expression beyond the time of XCI initiation, in contexts where it was not expected to initiate gene silencing [76]. Notably, this effect was shown to depend on SPEN, the key effector of Xist-mediated silencing at the onset of XCI during embryonic development, whose depletion leads to increased expression of escapees from the Xi [76,123]. Increased Xist RNA levels reduced the transcriptional activity of all escapees, but constitutive and facultative escapees responded differently: the former resisted full inactivation, whereas the latter became irreversibly silenced and acquired promoter DNA methylation upon prolonged Xist overexpression [76]. The difference in susceptibility to Xist RNA between facultative and constitutive escapees may reflect distinct regulatory mechanisms, with constitutive escapees likely having evolved gene-specific strategies that allow them to always evade Xist-SPEN-mediated silencing, whereas facultative escapees may be more or less prone to escape or become reactivated depending on the levels of Xist RNA encountered during XCI establishment.

Finally, another layer of X-linked gene regulation whose relevance to XCI escape remains unclear is X-chromosome up-regulation (XCU), the process by which genes on the Xa become up-regulated in the absence or inactivation of a second X chromosome to balance X-linked gene expression with that of the autosomes [124]. Recent evidence suggests that XCU is regulated on a gene-by-gene basis at both the transcriptional and protein levels [125]. In the case of escapees, a tendency for increased expression in X monosomy was observed in mouse ESCs, although it did not reach statistical significance, likely because of the small number of escapees analyzed [125]. Nevertheless, whether escapees, whose expression from the Xi is dampened rather than fully silenced, are subject to XCU remains little explored, as well as the extent to which Xa up-regulation may contribute to their overall RNA and protein dosage across different cellular states before and after XCI.

The studies described above underline the need for comparative and locus-specific approaches to dissect the complexity of escape regulation, define how X-linked dosage sensitivity is established and maintained across different sex chromosome complements, and determine whether escapee activity is controlled by distinct mechanisms across cellular and developmental contexts.

## Perspectives

- Deciphering how escapees are regulated will be essential for understanding their functions and for uncovering the (epi)genetic basis of sex differences in development and disease.
- Escape from XCI is more dynamic and context-dependent than initially appreciated, highlighting the importance of studying this process across organs and cell types to capture its full heterogeneity.
- Future studies will distinguish contexts in which escape from XCI is actively controlled from those arising due to defects in XCI maintenance and will clarify whether its biological effects are driven by individual gene activity or variation in heterochromatic Xi copy number.

## Open Access

Open access for this article is provided via our Subscribe to Open programme, made possible through the support of our subscribing institutions. Learn more here: https://portlandpress.com/pages/open_access_options_and_prices#conditional.

## Abbreviations

NPCs: neural progenitor cells
PAR: pseudoautosomal region
SLE: systemic lupus erythematosus
TAD: topologically associating domain
TF: transcription factor
Xa: active X chromosome
XCI: X-chromosome inactivation
XCU: X-chromosome up-regulation
Xi: inactive X chromosome
Xp: paternal X chromosome
Xm: maternal X chromosome
